# Diphtheria

**Published:** 1889-01-05

**Authors:** 


### DIPHTHERIA.

DIPHTHERIA, from διφθέρα, a skin, is defined in most standard works on medicine as a specific contagious disease, endemic in certain places, and sometimes prevailing as an epidemic, characterised by the formation in various situations, particularly in the throat, of a peculiar tough false membrane. This membrane is sometimes so tough that the disease has often been called "leather-throat." The term diphtheria was unknown in medical literature thirty years ago, and was first used by Mons. Bretonneau, of Tours. But diphtheria is not a new disease. There is no doubt that it existed in the time of Hippocrates, and at more recent periods medical authors were content to apply such terms as putrid sore throat, inflammatory sore throat, or croup, to the malady. Even now considerable difference of opinion exists as to whether diphtheria and croup are distinct diseases, the weight of evidence being, however, as we think, in favour of the affirmative reply. Opinions are also divided as to whether diphtheria is a general disease or a local malady. The latter view receives support from experiments on inoculation of the disease in animals, and from the fact of medical men and attendants on the sick having apparently contracted it by the direct conveyance of the *materies morbi* from the sick. Even animals, as cats or dogs, have been presumed to convey the affection, as referred to in the *British Medical Journal* of the 14th of July last. And it has been frequently noticed that diphtheritic affections are often co-existent with throat diseases of pigeons, fowls, pheasants, and other birds. Whether or not there is intimate connexion between these ailments in bipeds, quadrupeds, and birds, a consideration of the various views and writings on diphtheria leads to several practical conclusions. These are, First, that diphtheria is a malady communicable both by infection and by contagion: meaning by infection, transmission through the atmosphere, and by contagion, communication of *materies morbi*, directly from the sick, or indirectly by the media of articles of clothing, culinary utensils, or animals, etc. Secondly, that grave constitutional disturbance is a prominent symptom of severe cases of diphtheria. Thirdly, that certain important consequences or sequels are apt to follow diphtheria, especially some forms of paralysis.

As is the case with many other diseases, notably scarlet fever, typhoid fever, and small-pox, cases of diphtheria differ much as to intensity, from the mildest form, which resembles an ordinary catarrhal sore throat, to the most severe character, when the malady assumes a gangrenous or putrid form. This again leads to much difference of opinion; for, the throat affection which one physician might dignify by the name of diphtheria, another physician might designate by a more homely title. This variability of the affection also leads to very different estimates of mortality from it. It also results in physicans who consider all cases of throat disease with the least exudation to be diphtheria, being apparently much more successful in their treatment of the disease than others who do not apply the term so generally. It appears to us, however, that as diphtheria is so infectious and contagious, it is well to err on the safe side, even although the term diphtheria may be applied to a malady when the application may not be pathologically correct.

The symptoms of a typical case of diphtheria may be briefly mentioned as follows: First, there are characteristics of a common cold, such as chilliness and depression, while the throat feels uneasy and stiff. When examined internally, it is seen reddened and swollen. The glands at the angles of the lower jaw may also be enlarged. Soon the typical exudation appears on the inflamed surface, in the form of greyish white specks or patches with rosy-red border. These patches may remain separate, or they may spread, coalesce, and cover the whole surface. There may be no great amount of fever, but there is usually great prostration of strength; and about the third or fourth day of the malady, albumen is generally found in the urine. When recovery occurs the favourable signs are: arrest of the extension of the false membrane, and the detachment and expectoration of that already formed; improvement of the strength, and disappearance of albumen. Unless a favourable change occurs, death takes place in three or four days, either from the extension of the false membrane into the air passages, causing asphyxia, or from weakness and general collapse. But, as before observed, the symptoms vary much. When diphtheria gets into a house, if the inmates are living in good sanitary conditions it usually falls light; but if the reverse is the case, and especially if the drinking water happens to be contaminated from sewage or drains, the disease spreads with appalling rapidity and mortality. The disease is so frequently associated with defective sewage that it has also been termed *drain-throat*.

Judging from the numerous recommendations for the treatment of diphtheria, and from the many suggestions constantly appearing in the medical journals, it would seem that many physicians have a favourite method of their own. It is questionable, however, if success in the treatment of typical diphtheria does not depend more on the condition and age of the patient, and the hygienic conditions under which he is placed, than in the use of any medicine. There is, however, a consensus of opinion that warmth, good ventilation of the sick-chamber, and moistening of the air by a steaming kettle, are essential. Similarly all agree that the patient's strength must be supported by broths, soups, beef tea, and stimulants so long as the patients can swallow, and by nutritive enemata if swallowing is not possible. As medicine the tincture of perchloride of iron has the greatest number of advocates, either combined or not with chlorate of potash. Many, however, recommend quinine in preference. Topical applications to the throat are recommended by some; no local interference by others. For our own part we prefer a solution of carbolic acid, which may be used, if possible, as a gargle or otherwise introduced as spray, *not* with the view of detaching the membrane, but as a disinfectant. The forcible removal of the false membrane is to be condemned—although recommended by a continental authority, for it leaves a raw surface on which the exudation is quickly reproduced.

We desire, however, to call attention to a method of treatment recently very strongly advocated by Dr.

Murray-Gibbes, of Taranaki, New Zealand. In the *Australian Medical Journal* for July 15th last, Dr. Murray-Gibbes reported great success by the use of blue gum leaf steam (eucalyptus globulus). Dr. Murray-Gibbes states "constant blue gum leaf steam will cure diphtheria in all its forms." Dr. Murray-Gibbes also brought forward the experience of Dr. O'Carroll, who reported equally successful experiences; also that of Dr. Willis, of Daylesford, was very successful, although to a smaller extent. In the *Australian Medical Journal* of October 15th last there is a further report from Dr. Murray-Gibbes, read before the Medical Society of Victoria. From this it appears that the gentlemen named above have collectively treated 475 cases of diphtheria, with only two deaths. The method of treatment is thus described by Dr. Murray-Gibbes: 'I make a tent over the head of the patient's bed by placing an open umbrella with a sheet over it. On a chair or stool beside the bed is placed a jug, and in it is put a handful of blue gum leaves, and the jug is then filled with boiling water, and the whole is changed every half-hour in ordinary cases or every quarter of an hour in severe ones. The patient is thus kept in a warm, moist atmosphere night and day." No medicine internally, except a powder of *hydrargyrum cum creta* with scammony as an aperient, to be repeated as necessary. The author's theory is that an antiseptic agent is transmitted in the steam from the blue gum leaves, which destroys the microbes on which the malady depends. It is necessary that the leaves of the *Eucalyptus globulus* should be used, and *not* those of the *E. amygdalus*, which are inert. Oil obtained from the leaves has not a similar curative effect. In the discussion which followed the reading of Dr. Murray-Gibbes' paper, Dr. Maudsley, of Victoria, observed that he had seen the eucalyptol oil used at University College, London, and also leaves obtained from the London Botanical Gardens, without good result. Several other members observed that a mortality of one per cent. in diphtheria was utterly unprecedented in the history of medicine," but Dr. Murray-Gibbes persistently replied that "what he could say definitely was that the disease he saw in New Zealand gave way to the treatment he advocated."

While hoping the reverse, we fear the blue gum treatment of diphtheria will eventually be relegated to that limbo which has received so many reputed specifics for so many diseases. Iron, quinine, calomel, chlorodyne, salicylic acid, carbolic acid, chloride of sodium, sulphate of soda, chlorate of potash, copaiba and cubebs, sulphuret of potash, bromine and its compounds, *cum multis aliis*, have at one time or other been credited with repute as specifics or semi-specifics for diphtheria. Still, we call attention to the statements made by Dr. Murray-Gibbes in the hope that those medical men in a position to do so will be induced to try the plan of treatment advocated with such ardour by the Australian physician; who, however, we cannot help thinking must be classed with those practitioners designating all affections of the throat, mild or severe, as diphtheria.

